# Supplementary material for: Exploration of attitudes regarding uptake of COVID-19 vaccines among vaccine hesitant adults in the UK: a qualitative analysis
Source: BMC Infect Dis. 2022 Apr 26;22:407. doi: 10.1186/s12879-022-07380-9 (PMC9039596; doi:10.1186/s12879-022-07380-9)
Supplement: Supplementary file 1 — Additional file 1. Coding framework. [file 12879_2022_7380_MOESM1_ESM.docx]

Additional file 1: Vaccine hesitancy coding framework

| Theme | Code | Description |
| --- | --- | --- |
| Benefits of vaccination | | |
| Protect yourself and those around you | Protecting yourself | Includes comments from partially and unvaccinated participants who recognise that vaccination can protect themselves and those around them |
|  | Protecting vulnerable members of family | Includes comments from partially and unvaccinated participants who recognise that vaccination can protect themselves and those around them |
|  | Protect society | Includes comments about the role of the vaccine in protecting wider society |
| Avoid restrictions /facilitate return to normal | Facilitate travel | Comments about how vaccination can facilitate travel and/or avoid travel restrictions. Can be a reason for having been vaccinated, or a possible reason for being vaccinated in the future. Often seen as the wrong reason for vaccination. |
|  | Socialise with others | Including, but not limited to, vulnerable others |
|  | Employment requirements | Includes comments from participants who may have to be vaccinated for work purposes. |
|  | Return to “normal” | General comments about the role of the vaccine in facilitating a return to normal (including removal of all restrictions and infection control policies). |
| Barriers to uptake | | |
| Lack of need or benefits (complacency) | Perceived immunity | Including natural immunity and/or immunity |
|  | Perceived low risk from covid | Often due to being young and healthy. Also includes comments from people who had had (mild or asymptomatic) covid. |
|  | Vulnerable people protected through their own vaccine | A lack of understanding of the role their vaccine would play in protecting others (often linked to efficacy of the vaccine). |
| Effectiveness / efficacy  (Confidence) | Reducing transmission | Often linked to personal or statistical evidence to support that people can still catch and transmit covid once vaccinated |
|  | Reducing severity | Comments about the role of the vaccine in reducing severity |
|  | Against new/future variants | Comments about the efficacy of the vaccine against new or future variants. Also includes comments about how long protection from the vaccine lasts. |
| Safety  (Confidence) | Known side effects | Including blood clots and other side effects that participants had witnessed or experienced (personally or vicariously). May or may not be legitimate side effects. Particular weight given to personally relevant side effects (e.g., fertility for those wanting to become parents). |
|  | Unknown /future side effects | Includes comments about the potential for long term side effects that had not been identified. |
|  | Lack of research /long term follow up | Concerns about a lack of research, particularly with long term follow up periods. Also includes comments about the speed at which vaccinations had been created and developed (particularly in comparison to previous vaccination development studies). |
| Opportunity (convenience) | Perceived or experienced access barriers | Including being unable to take time off work to attend appointments and / or other reasons preventing physical attendance at a vaccination centre. |
|  | Location of vaccination centres | Includes comments about locality of centres and the importance of easy access/ familiarity. |
| Assessing risks and benefits | | |
| Assessing risks and benefits | Risks outweigh benefits | Any comments in which participants attempt to consider the benefits and potential harms of vaccination. |
|  | Benefits outweigh risks |  |
|  | Uncertainty |  |
| Factors influencing risks and benefits | | |
| Age and health status | Age reduces risk from covid | Impact of age on participants’ assessments of the risks and benefits of vaccination. |
|  | Age increases risk from side effects |  |
|  | Age linked to fertility concerns |  |
| Understanding of science | Understanding risk messages | Comments indicating (or demonstrating) confusion around risk messages and what they mean. Including (lack of) understanding why messages change. |
|  | Understanding and interpreting scientific evidence | Comments relating to (lack of) existing scientific evidence and inferences that can (and are) drawn. |
|  | Prioritising experience over statistics | Preference for visible over scientific evidence. |
| Trust in government | Mistrust of government | Includes comments about the integrity of the government and it’s handling of the pandemic. |
|  | Conspiracy theories / ulterior motives | Possible motives ulterior motives for vaccination (e.g., financial/ dictatorial). |
|  | Associated organisations | Includes comments about organisations (e.g., NHS/BBC) assumed to be under government control. |
| Experience and expectations | Experience of / beliefs about covid | Previous experience and belief systems influencing current attitudes to vaccination. |
|  | Experience of / beliefs about vaccines |  |
